# Infection control in the brain and the eye

**DOI:** 10.1111/aos.70071

**Published:** 2026-02-07

**Authors:** John V. Forrester, Paul G. McMenamin, Samantha J. Dando

**Affiliations:** ^1^ School of Medicine, Medical Sciences and Nutrition, Institute of Medical Sciences University of Aberdeen Aberdeen UK; ^2^ Monash University Melbourne Victoria Australia; ^3^ Neuroecology Group, School of Agriculture, Biomedicine and Environment La Trobe University Melbourne Victoria Australia; ^4^ Centre for Immunology and Infection Control, School of Biomedical Sciences, Faculty of Health Queensland University of Technology Brisbane Queensland Australia

**Keywords:** CNS, immune privilege, infection, latency

## Abstract

The Central Nervous System (CNS), comprising the brain and the eye, is considered to have a ‘privileged’ mechanism for dealing with immunological challenge (immune privilege, IP). CNS IP has been revealed through experiments using foreign protein antigens and cell and tissue alloantigens (grafts), but evidence for a role for IP in modulating host–pathogen interactions in the CNS is limited. However, the low frequency of CNS infection in the face of widespread systemic exposure to CNS‐tropic infectious agents, together with the high incidence of CNS infection in immunocompromised individuals, suggests that in healthy individuals, the CNS has tightly controlled regulatory mechanisms to protect against infectious agents. Although the naïve healthy brain and retina parenchyma largely lack adaptive immune cells, their border tissues (meninges, uveal tract) contain a full complement of resident immune cells, including CNS‐specific regulatory T cells (Tregs), which have a fundamental role in controlling infection in the brain parenchyma. Tregs also underpin ocular IP, particularly of the neural retina. Recent studies report that Tregs are transcriptionally ‘customised’ to the CNS and function at a distance; that is, are located in niches/hubs around the venous sinuses of the border tissues. T cells resident in the uveal tract probably play a similar role. We propose that Tregs are key drivers of CNS IP and do so by promoting latency of infectious agents.

## 1 INTRODUCTION

‘Immune privilege’ (IP) as a concept began its life in the eye and the brain. In essence, the failure of the eye to reject foreign grafts (skin allografts), a phenomenon which ran counter to the notions of self‐nonself discrimination (SNSD) and immunological tolerance (IT) (reviewed in Park, 2006), led to the concept of IP (Billingham & Boswell, 1953). Indeed, Medawar's early experiments were in part an attempt to understand the mechanism behind the long‐held recognition of successful acceptance of corneal allografts (Crawford et al., 2013). IP has since been implicitly expanded to describe the CNS response to foreign antigens generally (Zanluqui & McGavern, 2024) but evidence for how IP applies to infectious agents is limited. It is true that infections in the CNS are uncommon and those that do occur are considered to be a ‘cost’ of IP (Caspi, 2006) but direct experimental evidence for IP to pathogens in the CNS is lacking (Molzer et al., 2020).

IP is a time‐honoured notion, almost going back to the beginnings of Immunology as a discipline. Originally reported in 1873 by van Dooremaal (cited in ref., Niederkorn, 2006) and firmly embedded within the immunological canon following Medawar's allograft experiments (Medawar, 1948), IP has been viewed in somewhat ‘black and white’ terms. As indicated above, IP was also reported to apply to allografts in the brain (Medawar, 1948) and so was thought to be a signature feature of the CNS immune response to foreign antigen. However, the reliability of these experiments has been challenged (Barker & Widner, 2004). In addition, Perry's group (Galea et al., 2007) and others observed that IP was not absolute, but could be expressed to different degrees by different tissues. Matzinger (Matzinger & Kamala, 2011) took this further and suggested that tissues themselves generally determined the strength and class of the adaptive immune response.

Arguments have gone back and forth on whether IP is fundamentally different from the wider concept of immune tolerance (IT). Studies of immune responses in dizygotic twin cattle (Owen, 1945) and materno‐foetal rhesus blood group disparities in humans (Owen et al., 1954) contributed to the theory of SNSD, which became the touchstone of IT (Medawar, 1961). IP was proposed as an explanation for phenomena which were out of kilter with the SNSD‐based concept of IT, and definitions of both IT and IP have been revised and refined on several occasions (Carson & Wilson, 2013; Caspi, 2006; Devi & Anandasabapathy, 2017; Gery & Caspi, 2018; Howie et al., 2014; Margo & Harman, 2016; Mellor et al., 2017; Takenaka & Quintana, 2016; Waldmann, 2010; Yin et al., 2021).

SNSD remains a central tenet of IT. However, IT can be applied not only to self‐antigens but also to any foreign antigen, whether recognised by the immune system as pathogenic, commensal, symbiotic or mutualistic (Ayres & Schneider, 2012). This conceptual shift aligns well with Matzinger's danger hypothesis (Matzinger, 2002). IP in essence equates to IT, in part because it uses the same immunological mechanisms as IT. These include deletion of T cells (Ferguson & Griffith, 2007; Yin et al., 2015), induction of T cell anergy (Streilein et al., 1997) and induction of T regulatory cells (Gregerson et al., 2009; McPherson et al., 2014). However, in IP, the focus shifts from what is happening to the T cells towards the role of the tissue in directing the changes in T cell behaviour.

As we have argued, IP in one sense is a property of all tissues to a greater or lesser degree (Forrester et al., 2018; Molzer et al., 2020). In addition, the assumed teleological value of IP in the context of challenge by pathogens is unclear: is the host ‘weighing up’ the risks from failing to get rid of the pathogen against the pathogen's potential for inducing life‐threatening tissue damage (Caspi, 2006)? Indeed, ‘privilege’ is probably a misnomer for a process whereby the immune system is ‘customised’ by the tissue to balance pathogen neutralisation with limiting tissue damage. Here, we discuss how the CNS parenchyma responds to challenge by infectious agents.

## 2 THE CNS IMMUNE SYSTEM

All tissues have properties which maintain immune homeostasis, including fluid drainage systems and populations of resident immune cells which act as scavengers (for waste removal) and sentinels (for ‘danger’ sensing). Interstitial fluid in the brain is limited compared to other tissues but is actively drained bidirectionally depending on local pressure gradients, between the CSF, the arterial and venous blood streams and the meningeal lymphatics within the dura mater via the conduit glymphatic system (reviewed by Bohr et al., 2022). CNS antigens, shed through normal cell physiology in the brain (Harris et al., 2014) and the retina (Voigt et al., 2017), are drained to the deep cervical lymph nodes (Louveau et al., 2015; Yin et al., 2024) through this system as well as through the blood stream to other tissues such as the thymus and the spleen after first pass through the pulmonary circulation and the lungs (Vendomele et al., 2017; Wang et al., 1997). Indeed, the glymphatic network has been described as the brain's waste removal system and is most active during sleep, particularly non‐REM sleep (Bohr et al. 2022) while a similar system has been observed in the retina/optic nerve (Wang et al., 2020). Mathematical studies of fluid efflux from the brain estimate a velocity of 60–190 mm/h (Bork, Hauglund, et al., 2024).

**FIGURE 1:**
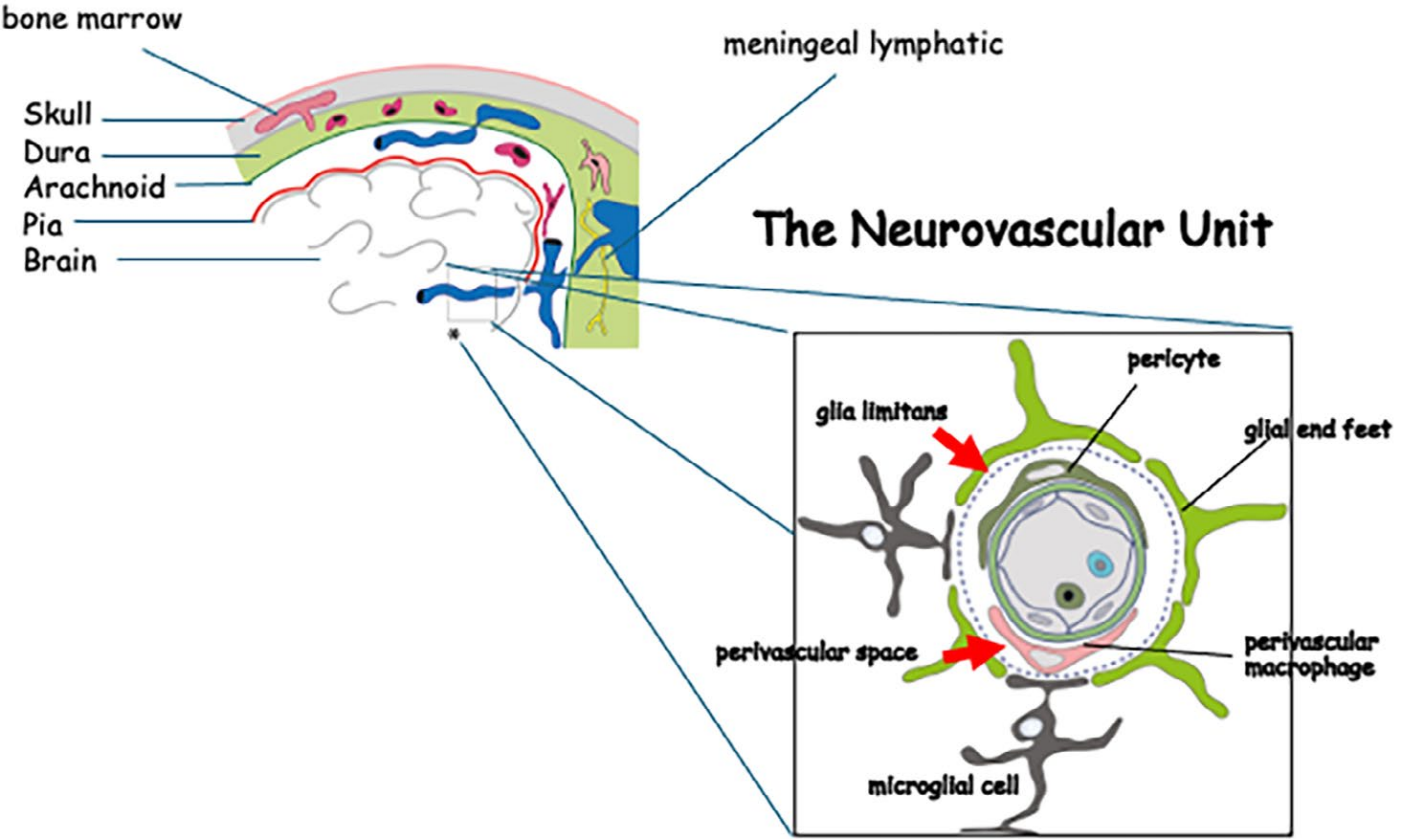
Organisation of the meningeal immune system showing the dural andleptomeningeal (arachnoid, pia) border regions covering the brain parenchyma. These layers extend a continuous sheath surrounding the blood vessels as they penetrate into the brain and are lined externally by the glia limitans serving as a third barrier against passage of cells and proteins into the brain parenchyma. The perivascular space is continuous with the subarachnoid space and contains a range of immune cells. The dural layer also contains T and B cells in "hubs" centred around the venous sinuses.

A sophisticated three‐layered system of functional ‘barriers’ insulates the CNS parenchyma from the immune system proper under healthy conditions, limiting access by immune cells and effectors such as immunoglobulins (for review see ref. Buckley & McGavern, 2022). The blood–CSF (BCSF) barrier, the blood–brain barrier (BBB) and the glia limitans/glia perivascularis (GL), formed by astrocyte foot processes, are the barriers in the brain, and the blood aqueous barrier (BAB), the blood retinal barrier (BRB) and the Muller cell‐supported glia perivascularis (GPV) are the barriers in the retina. In both the brain and the retina, the GL/GPV barrier forms the outer layer of the capillary neurovascular unit (NVU) (Obermeier et al., 2016; Figure 1).

A population of resident immune cells exists in the meningeal and uveal stroma internal to the BCSF and BAB, respectively (Dando et al., 2019; Forrester et al., 2010; McMenamin et al., 2003; O'Koren et al., 2019). Richly endowed with macrophages, dendritic cells and neutrophils (Patel et al., 2025), these ‘border tissues’ also contain small collections of other innate immune cells such as NK cells (inducing TRAIL‐expressing immunoregulatory astrocytes; Sanmarco et al., 2021), MAIT (Mucosal‐Associated Invariant T) cells (Klein, 2022) and connective tissue mast cells (Patel et al., 2025) with roles in CNS immunoregulation (see below). In the meninges, T cells predominantly localise in the vicinity of the venous sinuses and include T resident memory cells (TRMs) (Netherby‐Winslow et al., 2020) and T regulatory cells (Tregs) (Liston et al., 2022; Marin‐Rodero et al., 2025). B cells are similarly distributed and have tissue‐specific properties, as are the T cells (Brioschi et al., 2021; Fitzpatrick et al., 2024). B cell access to the meningeal niches appears to be directly derived from skull bone marrow (Brioschi et al., 2021). Interestingly, there are also small numbers of innate lymphoid cells (ILCs1, 2 3) (Wang & van de Pavert, 2022) in the meninges of which ILC3's may have a role in maintaining the Treg population (Ahmed et al., 2024). Finally, a small population of plasma cells also lines the meningeal vessels which predominantly secrete IgA and appear to be ‘educated’ in the gut before tracking to the meninges. This provides a protective barrier to invasion of the cerebral parenchyma by fungi such as *Candida* spp. (Fitzpatrick et al., 2020). Collectively, the congregation of immune cells in the dural meninges has been described as the dura‐associated lymphoid tissue (DALT) (Abedalthagafi et al., 2009), one variation of which takes the form of rostro‐rhinal lymphoid hubs (Fitzpatrick et al., 2024). Recently, a fourth meningeal layer has been described in the mouse which partitions the subarachnoid space (the subarachnoid lymphatic‐like membrane [SLYM]), which acts as a mesothelium‐like covering and is thought to be the murine equivalent of the arachnoid granulations in humans transporting fluid and low molecular weight solutes (Mollgard et al., 2023).

It is not clear whether the ‘resident’ population of immune cells in the CNS border tissues has roles in protection against infection, in immunoregulation or both (see below). Furthermore, life‐threatening pathology may not directly be due to infection. For instance, during infection of the CNS with lymphocyte choriomeningitis virus (LCMV), meningeal CD8 T cells recruit large numbers of myelomonocytic cells from the bone marrow which invade the meninges and cerebrum and act as the main tissue‐damaging cells. This leads to herniation of swollen brainstem tissue which is the immediate cause of death (Kim et al., 2009). More commonly, however, in homeostasis, the resident, yolk sac‐derived myeloid cells which populate the meninges and the uveal tract are mainly considered to be tolerising/suppressor cells. These cells have recently been identified as the conveyers of CNS self‐peptides which promote tolerance, prevent autoimmunity and act as the guardians of IP (Kim et al., 2025).

As indicated above, the complement of immune cells in the CNS border tissues is large and varied. Most of these are absent from the brain parenchyma and the retina. However, an important yolk sac‐derived, self‐renewing myeloid cell type, namely the microglial cell, is widely distributed throughout the brain and retina, with connections to most neural and supporting glial cells. The main role of microglia is one of tissue homeostasis with emphasis on synaptic plasticity and trimming of neuronal connections rather than as primary CNS immune cells; microglia also change extensively over time from development to aging (discussed in refs. Kettenmann et al., 2011; Tay et al., 2017). A relatively small cell with many dendritic processes, microglia not only participate in CNS immune responses, with a primary role in maintaining CNS immune tolerance (see next section) (Hong et al., 2023) but also acting in a proinflammatory manner in states of inflammation, for instance in neurodegenerative diseases (Rao et al., 2024) or in fungal invasion of the CNS (Reyes & Shinohara, 2022). Activated microglia lose their processes and attachments to other cells and become rounded and migratory during CNS inflammation while a significant role in antigen presentation is controversial. A comparative review of border tissue macrophages and parenchymal microglia has recently been reported (Van Hove et al., 2025) but the role of microglia in CNS IP is unclear (Forrester et al., 2018).

## 3 IMMUNOLOGICAL TOLERANCE (IT) IN THE CNS

IT is the default process whereby the organism recognises, but does not respond to, ‘harmless’ antigens, including commensal antigens of the microbiome (Shao et al., 2023) and most self‐antigens. This process is mediated through presentation of antigen by conventional DCs (cDC), particularly Type1 cDC (cDC1), to T cells and involves generation of various types of regulatory cells including anergic and regulatory T (Kalekar & Mueller, 2017; Rouhani et al., 2015) and B cells (Dufaud et al., 2018; Rosser & Mauri, 2015).

cDC maintain IT provided the circumstances are right, and the strength and quality of the immune response is modulated by the tissues in which the DC reside (Matzinger & Kamala, 2011). In the CNS, IT is maintained by the continuous shedding of antigens either as soluble or as cell‐associated antigen (after phagocyte uptake), which drain constitutively to the cervical lymph nodes as part of normal physiology (Aspelund et al., 2014; Mohammad et al., 2014; Shibata‐Germanos et al., 2020). Cell‐associated antigen derives from apoptotic cells which have been engulfed as part of normal tissue cell turnover as well as from shedding of cellular proteins into the extracellular matrix. Antigen trafficked to the cervical lymph nodes from the brain subserves IT mainly via Treg induction (Mohammad et al., 2014).

The scale of total antigen shedding into the interstitial fluid and transport to the DLN on a daily basis is vast: estimates of specific protein concentrations are in the nanomolar range (Clement et al., 2010), well within the level to initiate cDC antigen processing and presentation. Consequently, antigen presentation by tolerogenic (tol) DC to naive T cells, thereby generating regulatory/anergic T cells, becomes the major immune default function of the organism. This has special relevance to CNS IP (ref Peng et al., 2025 and see below).

**FIGURE 2:**
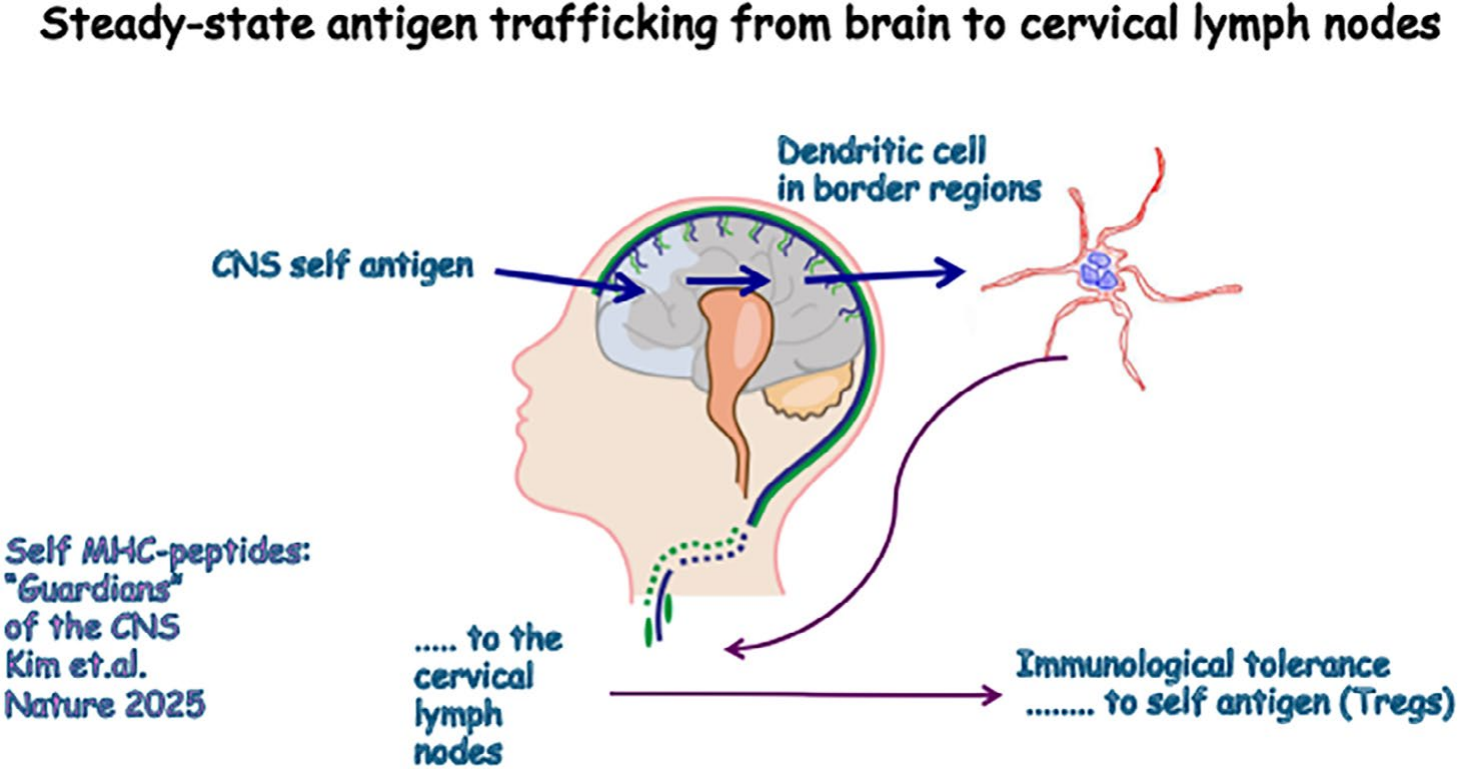
Diagram outlining induction of CNS tolerance to self antigens. Parenchymal brain cells shed proteins and peptides as part of normal cell physiology, which traffic through the interstitial fluid / glymphatic system to the border tissues surrounding the brain and the eye. cDC‐associated or soluble peptides then drain either into the dural lymphatics and on to the deep cervical lymph nodes, or into the CSF and venous system / spleen where they induce antigen specific immune tolerance (IT).

This long‐standing and widely held view of how IT generally is induced has recently been investigated in the context of CNS antigens. Kim et al. (2025) have identified an endogenous repertoire of CNS‐derived ‘regulatory’ self‐peptides bound to MHC Class II rather than Class I, which traffic from the brain, through the meningeal lymphatics to the draining cervical lymph nodes (Figure 2) and expand a population of regulatory CD4(+) T cells controlling neuroinflammation via activity of CTLA‐4 and TGFβ. Potentially, MHC II‐peptide complexes travel within extracellular vesicles shed from antigen presenting cells or as cell‐associated membrane cargo within the migrating cell. Shed soluble proteins may also traffic to the lymph node to be taken up and processed by LN DC thus promoting tolerance. These endogenous peptides were described as the ‘guardians’ of IP but, in reality, represent an elegant example of constitutive, homeostatic IT as it applies to the CNS.

## 4 HOST–PATHOGEN INTERACTION IN THE CNS

Most of the above knowledge regarding the CNS immune system has been captured in models of neuroinflammation, often involving autoimmunity. These include experimental autoimmune encephalomyelitis (EAE) (Berg et al., 2024) using endogenous purified CNS protein such as myelin basic protein (MBP) or myelin oligodendrocyte glycoprotein (MOG), and experimental autoimmune uveoretinitis (EAU) (Klaska & Forrester, 2015) using interphotoreceptor retinol binding protein (IRBP). There is also a very large body of work describing clinical, pathological and experimental studies on pathogens which infect the CNS and cause life‐threatening and sight‐threatening disease. All types of pathogens have been studied including viruses, bacteria, fungi, parasites and prion‐like agents. Most of these studies have concentrated on how the pathogen exerts its deleterious effects with the aim of trying to develop ways to limit damage. However, clinically overt CNS infection is rare. Indeed, as indicated above, epidemiological studies, often based on serology, show that most individuals exposed to and infected by these pathogens do not develop disease, or only develop mild transient symptoms. Examples include tuberculous meningitis, toxoplasmosis, cysticercosis, cryptococcosis, West Nile fever, dengue fever, herpes simplex, Epstein Barr virus infection and many more (reviewed in Forrester et al., 2018, 2022). Whether serious disease occurs depends on the host/pathogen interaction, which is a complex relationship.

### 4.1 What is a pathogen?

The concept of microbes as disease‐causing agents, that is, ‘pathogens’, emerged from ideas concerning air‐ and water‐borne small particles (pollutants) proposed many years before ‘germ theory’ was definitively articulated by Koch and Pasteur in the 19th century (Mendelsohn, 2002). The critical observation made by Koch and Pasteur was that the particles were living (‘germs’) and could proliferate in vitro and in vivo. Koch described four criteria (Koch's postulates) required for attribution of pathogen status to a microbe (Antonelli & Cutler, 2016; Byrd & Segre, 2016; Segre, 2013). Several pathogens were clearly identified, but even at that time, Koch recognised that many living microbes did not cause disease. Now we accept that almost all microbes that humans interact with are non‐pathogenic and most infectious diseases are caused by a limited number of microorganisms. For instance, around 50% of all bacterial infections are caused by only 20 species of bacteria (Callaway, 2025). Furthermore, many disease‐causing microbes (pathogens) only become pathogenic in the right context (opportunistic infections) and normally reside on the skin and mucosal membranes as commensals (Methot & Alizon, 2014). This is emphatically illustrated by the trillions of microbes constituting the endogenous microbiota (Human Microbiome Project, 2012).

Moreover, the pathogenesis of infectious diseases is not predetermined: it is the result of the specific host–microbe interaction. Pathogens typically have an arsenal of virulence factors; however, damage to the host may not necessarily be the direct result of pathogen properties but is in part determined by the strength of the host response (usually the immune response). A weak response, as in immunodeficient patients, will allow the pathogen to replicate extensively and cause widespread cytolytic damage (tissue necrosis); alternatively, an uncontrolled host immune response (a failure of immune regulation) can also damage the host, either directly or through functional impairment or metabolic dysregulation. The cytokine storm of meningococcal meningitis and the dysregulated immune response of SARS‐CoV‐2 infections are but two examples. Koch's criteria, which defined a pathogen (Koch's postulates, reviewed in refs Antonelli & Cutler, 2016; Byrd & Segre, 2016; Segre, 2013), were useful in the time of pathogen discovery but do not stand up to scrutiny as new information accrues, such as carrier status or polymicrobial infections.

Most discussion on host–pathogen interactions reduces the host element to the common denominator of a predictable and reproducible host response. However, the host response to the same pathogen varies greatly from tissue to tissue. For each organism, this can range from undetectable without clinical signs (asymptomatic) to severely tissue‐destructive and life‐threatening. Typical examples include *Streptococcus pneumoniae*, which is a common commensal but can cause severe meningitis or pneumonia (Narciso et al., 2025). Similarly, *Staphylococcus epidermidis* has opportunistic properties, which can be fatal (Meric et al., 2018). As a consequence, it has been suggested infectious agents should be considered pathogenic when the host–microbe interaction results in tissue damage or death (Casadevall & Pirofski, 2015) and can be expressed within a Damage Response Framework (DRF) (Casadevall & Pirofski, 2003, 2015; Pirofski & Casadevall, 2008).

### 4.2 What is a host?

Just as a pathogen cannot be described in the absence of a host, a host cannot be identified unless it is host to infectious agents. Thus, in biological terms, a host is an entity that houses an endogenous microbiota, interacts with microbes and results in ‘damage, benefit or indifference’ (Casadevall & Pirofski, 2015). As Casadevall and Pirofski have stated, this has led to a range of outcomes of the host–microbe interaction and includes states of ‘symbiosis, colonisation, commensalism, latency and disease’ (Casadevall & Pirofski, 2015). Restating the above, the outcome depends on both the host and the microbe and is a measure of the host defense (immune) response to the microbe, modified by the properties of the host tissue.

Does the CNS host microbes and have an endogenous microbiota? Although there are suggestions that the healthy brain and/or its coverings, as well as the neural retina, contain live microorganisms, the evidence is based on metagenomics and the risk of contamination in sample preparation, despite assiduous precautions, will always be a caveat to these data (Editorial, 2025; Link, 2021). Although distant from the CNS, the gut microbiome is recognised to have both beneficial and potentially deleterious effects on the healthy brain and eye, as well as on neurological disease and degeneration. For instance, a healthy gut microbiome is required for the development of the blood–brain barrier (BBB) amongst other important neural development processes (Macpherson et al., 2023). The gut–brain axis is a two‐way interaction whereby immune cells from the gut have been reported to traffic to the brain while signals arising in the brain find their way to the gut mesenteric neural plexus and modulate the gut microbiota and function. Most recently, in elegant studies of gnotobiotic mice, their intrinsically altered behavioural patterns (anxiolytic behaviour) were successfully treated by adoptive transfer of dendritic cells. It has been suggested that bacterial components, contained within CD11b+CD11c+CD103+ migrating gut DC delivered directly to the brain, constitute one method whereby microbiome and brain signals have a beneficial connection (Philip et al., 2025). However, this is counter to the conventional migration pattern of DC which typically occurs from the periphery to secondary lymph tissues where their journey terminates with presentation of antigen to T cells. In contrast, deleterious effects of the gut microbiome appear to play a negative role in some neurological diseases. For instance, etoxin‐secreting *Clostridium perfringens* colonises the bowel of multiple sclerosis patients. In experimental models, these organisms are associated with a loss of brain IP (Ma et al., 2023). In addition, specific gut taxa have been associated with pre‐clinical Alzheimer's disease (Ferreiro et al., 2023). In addition to the brain, for several years the relationship between microbiome and the eye has been studied. Dysbiosis has been shown to impact a range of eye disorders including macular degeneration, diabetic retinopathy and uveitis, while the potential beneficial effect of short‐chain fatty acids produced by the microbiota has been suggested (reviewed in reference Floyd & Grant, 2020).

Direct translocation of bacteria to the retina has also been reported in a model of retinal neurodegeneration (Peng et al., 2024), but the technical caveat mentioned above holds. However, the role of the gut in homeostasis of the CNS is generally one of promoting quiescence as shown by ‘gut‐educated’ NK cells promoting TRAIL expression on CNS astrocytes (Sanmarco et al., 2021).

Belkaid has stated that microbes ‘set the immunological tone of tissues’ (Ansaldo et al., 2021; Belkaid & Harrison, 2017) and in simple terms this depends on (a) whether the microbe is a pathogen, a commensal/symbiont or has become latent with the potential to reactivate; and (b) whether the host mounts a strong response and clears the pathogen, a weak response leading to latent or persistent (chronic) infection or no response in which the host treats the microbe as a commensal.

## 5 THE AIDS EPIDEMIC AND THE CNS HOST/MICROBE INTERACTION

People with HIV+ status that progress to acquired immunodeficiency syndrome (AIDS) are vulnerable to a range of opportunistic infections and infection‐related tumours. Susceptibility to infectious agents is directly related to low circulating CD4 T cell counts (a WHO diagnosis of advanced HIV disease requires a lymphocyte count of <200 CD4+ T cells per mL, Ford et al., 2025) and, untreated, infections become invasive and are often fatal. Death may be from HIV itself but more commonly is the result of opportunistic infections. Similar infections occur in non‐HIV‐infected immunocompromised patients (Pruitt, 2021) further emphasising the central role of immune protection.

CNS infections in particular account for a sizeable proportion of deaths in HIV patients and include diseases such as progressive multifocal leukoencephalopathy, cryptococcal meningoencephalitis, *Toxoplasma* encephalitis, *Angiostrongylus* eosinophilic meningitis, HIV encephalitis, cytomegalovirus encephalitis, neurosyphilis, tuberculous meningoencephalitis, *Histoplasma* encephalitis and varicella‐zoster virus meningitis (Le & Spudich, 2016). This represents a wide range of ‘opportunistic’ infections by microbes which transmit widely in the community but cause minimal or no symptoms in immunocompetent hosts. All classes of infections are included, some of which are regional, endemic infections such as cysticercosis (Moyano et al., 2016) and listeriosis (de Noordhout et al., 2014), while others are widely distributed involving large sections of the population. For instance, around a quarter of the world's population has been infected by *Mycobacterium tuberculosis* (World Health Organisation, 2025) and a third by *Toxoplasma gondii* (Montoya & Liesenfeld, 2004), but the majority of infections in both diseases are asymptomatic and deaths infrequent unless the infections occur in immunocompromised individuals. A mildly acute or asymptomatic infection in childhood or early adulthood passes without incident. The organism becomes undetectable but many months or years later reappears, often at a different site and frequently when the individual is immunologically impaired, stressed or elderly. Such infections in later life are sometimes considered new or re‐infections, but are more likely due to reactivation of an existing dormant or latent infection. This is the case with infectious agents which range from highly prevalent to almost universal in human hosts such as herpes simplex virus (Tang et al., 2024; Whitley, 2006) Epstein barr virus (Soldan & Lieberman, 2023) *Candida* spp. (Snarr et al., 2020) or *T. gondii* (Milne et al., 2020). Others are restricted to discrete populations such as *Taenia solium* (Moyano et al., 2016), or *Cryptococcus* spp. where childhood exposure to the fungus approaches 70% but CNS infection is rare but frequently fatal (Alanio, 2020; Goldman et al., 2001).

## 6 IS THERE A PATTERN TO THE CONTROL OF INFECTION AND ITS MANIFESTATION IN THE CNS?

Infections of the CNS typically occur due to incomplete clearance of the microbe from the initial site of infection in the periphery. In the CNS, the first obstacle to microbe invasion is the border tissue (the meninges, the uveal tract) where early control by phagocytes and innate lymphoid cells is followed by microbe‐specific T effector cells (Teff) (Paskeviciute et al., 2023) generated in the peripheral lymph nodes to clear the infection (Forrester et al., 2018). This process, if uncontrolled, can lead to immune‐mediated damage (cytokine storm), which is particularly damaging to the CNS (Ayres & Schneider, 2012; Gadani et al., 2015; Klein & Hunter, 2017; Martins et al., 2019; Pirofski & Casadevall, 2008). Recently, it has been shown that the border tissues house CNS‐specific resident Tregs located in hubs around the meningeal venous sinuses (Marin‐Rodero et al., 2025) which give it immune privileged status. Meningeal Tregs are more numerous than the equivalent population in the spleen, form small perivascular clusters around cDC2‐type DC and prevent Teff cell access to the brain parenchyma (Marin‐Rodero et al., 2025; O'Brien et al., 2017). Furthermore, the meningeal Treg population is heterogeneous, including Th1‐like Tregs, T follicular regulatory cells (Tfr cells) and quiescent (unactivated) Tregs. Overall, meningeal Tregs appear to control the endogenous population of CNS T cells in the steady state and can expand when the CNS is threatened (Marin‐Rodero et al., 2025). Similar tissue‐specific Tregs in other tissues such as skin, liver, gut, white adipose tissue and bone marrow provide to each tissue its own flavour of ‘privilege’ (or not), and reflect how each responds to any particular pathogen (Fujisaki et al., 2011; Panduro et al., 2016).

The concept of Tregs as agents of IP is not new. In the eye, Gregerson's group (Gregerson et al., 2009; McPherson et al., 2011, 2013, 2014) and Keino et al. (2018) (reviewed in ref. Keino et al., 2018) have provided strong evidence that Tregs underpin ocular IP and suggested that they can be generated and induced to act locally. However, it is not clear whether induced ocular Tregs are derived from a resident, uvea‐specific population as occurs in the meninges (Marin‐Rodero et al., 2025) or are recruited from a circulating pool as part of the inflammatory process. Small populations of tissue‐specific T cells occur in the uveal tract (Etebar et al., 2024), but whether this includes Tregs has not been shown. However, since it has been proposed that Tregs, despite expressing a core Treg transcriptome, have tissue‐specific signatures, it is likely that they exert different types and levels of regulation in one tissue such as the skin or gut, and another in the CNS. For instance, Tregs in the gut signal through PPARγ and are educated by the microbiome (Busbee et al., 2020), while in muscle and visceral adipose tissue (VAT), Treg IL‐10 seems to play a counter‐intuitive role (Beppu et al., 2021; Villalta et al., 2014). In the CNS, the IL33:ST2/amphiregulin (Liston et al., 2022; Prasad et al., 2023) axis may be more relevant (Xie et al., 2022; Yao et al., 2025) and has been shown to have an immunomodulatory effect in a range of retinal diseases (Barbour et al., 2014; Scott et al., 2021; Theodoropoulou et al., 2017).

## 7 TREGS AS MEDIATORS OF CNS IMMUNE PRIVILEGE

**FIGURE 3:**
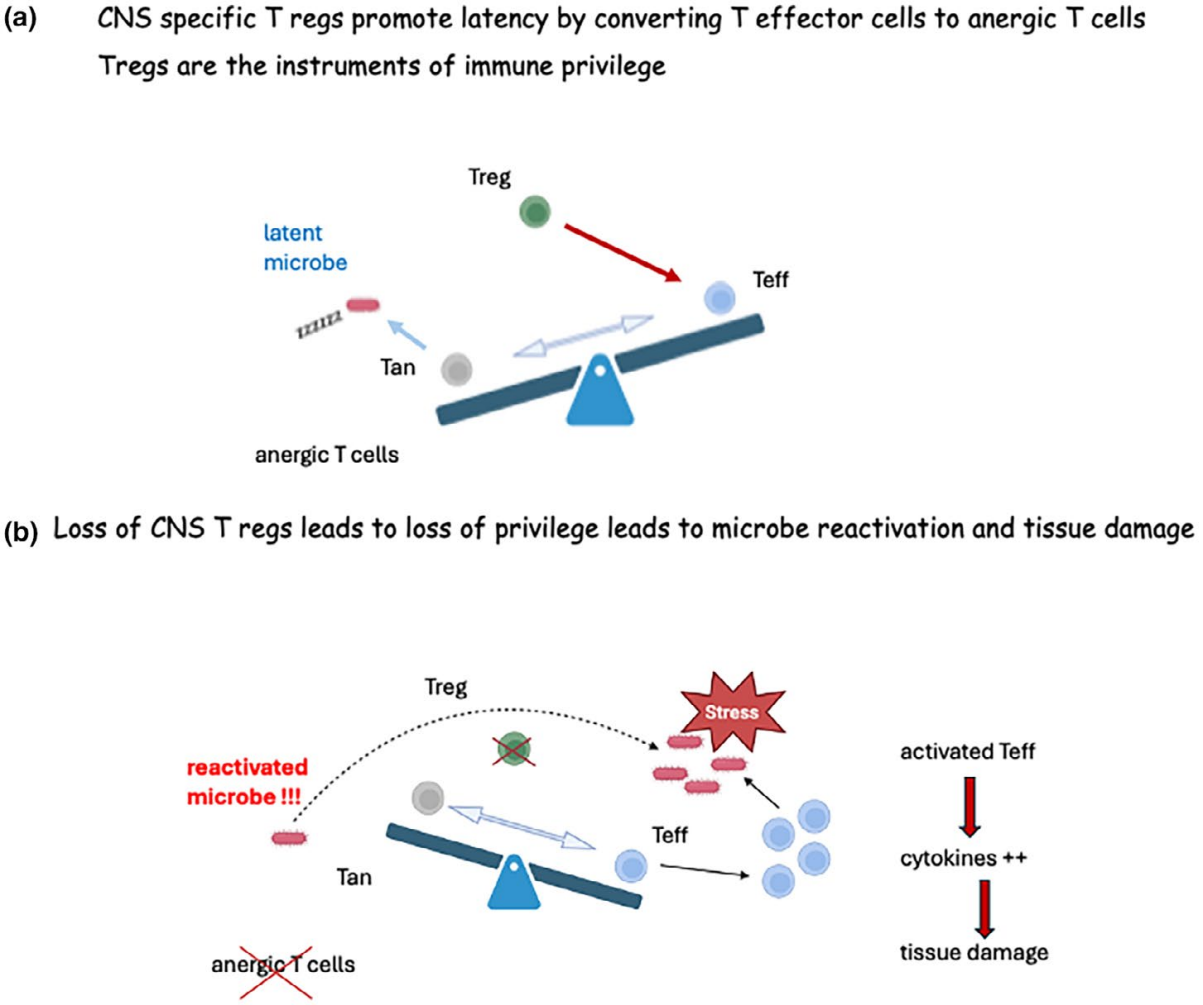
Tregs present in the CNS are instruments of immune privilege (IP) (Kim et al. 2025) and may provide IP‐mediated protection during infection by inducing latent infection. (a) In quiescence,Tregs act on Teff cells to promote anergy and limit Teff pro‐inflammatory effect, thereby facilitating microbial latency. (b) Loss of Treg control during stress or immunodeficiency allows microbial proliferation and increased levels of infection with tissue damage and /or increased Teff function leading to severe inflammation, cytokine storm and tissue damage (Figure created through Biorender).

CD4+ Tregs exist as two broad categories: FoxP3+ CD4+ Tregs and FoxP3‐CD4+Tr1 cells. They mediate their function by multiple mechanisms. The transcription factor FoxP3 characterises the most well‐recognised Tregs but is not essential for its immunoregulatory function. Instead, FoxP3+ cells regulate immunity through direct and indirect means: directly, by consuming IL2 required for survival by Teff (Tran et al., 2009), by secreting IL10 and TGFβ (Sakaguchi et al., 2020), by acting through co‐inhibitory molecules such as CTLA4 (Ahn et al., 2025) and PD‐1 (Musial et al., 2024), ST2‐3/amphiregulin (Barbour et al., 2014; Liston et al., 2022), alpha MSH and Fas/FasL (reviewed in Taylor & Ng, 2018), while indirectly Tregs inhibit antigen presentation by DC and macrophages through contact mechanisms (reviewed in Sakaguchi et al., 2020). Expression of FoxP3 in Tregs appears to be under the control of the gene repressor complex RBPJ‐NCOR rendering the gene somewhat unstable (Chen et al., 2025). In addition, FoxP3+ Tregs also have a role in tissue repair including the CNS (Constantinides & Belkaid, 2021; Malko et al., 2022; Xie et al., 2022). Several Treg mechanisms of action overlap with those attributed to CNS IP (Stein‐Streilein & Caspi, 2014; Taylor & Ng, 2018) and it is likely that CNS Tregs are the source of this immunosuppressive activity. This is not to disclaim the evidence of immunomodulatory factors contributed by endogenous CNS cells such as the IL33/Amphiregulin axis in non‐immunocyte CNS cells including astroglia, choroid plexus epithelia and RPE cells (Fairlie‐Clarke et al., 2018). These cells have been identified as sources of CNS IP, but since CNS Tregs can produce all of the same mediators and can be regarded as the ‘professional’ immune regulators, the constitutive presence of CNS specific Tregs supports a view that these cells are major mediators of CNS IP. In the eye, in particular, IL33 has been shown to prevent the development of EAU and retinal degeneration (Barbour et al., 2014; Scott et al., 2021; Theodoropoulou et al., 2017). Interestingly, a recent report has provided evidence that the ocular microenvironment may be conducive to the generation of eye‐specific FoxP3 Tregs. Peng et al. have shown that naive antigen‐specific T cells introduced into the eye become primed to retinal antigen. Around 30% of the primed cells differentiate into FoxP3+ Tregs while the remaining cells become anergic (Peng et al., 2025). This relationship of Treg cells to anergy is well known and has been shown to be active in Tregs used to prevent spontaneous ocular inflammation (Liu et al., 2020; Figure 3).

In contrast, Tr1+ T cells have not been described in the same context in the CNS, although aspects of IP have been attributed to Tr1 activity. Less is known about the ontogeny of Tr1 cells. They arise in the periphery from naive T cells, or from Th1 and Th17 memory T cells, through induction by tolDC via mediators such as vasoactive intestinal peptide (VIP) and pituitary adenylate cyclase‐activating polypeptide (PACAP) (Delgado et al., 2005; Lee et al., 2021). Tr1 cells are specialised in expression of LAG3 and CD49b and production of high amounts of IL10. In addition, pathogens invading the brain may generate brain‐endogenous PACAP, which is a recognised indirect mediator of IP (Lee et al., 2021) by downregulating excessive Teff responses. It is not known whether Tr1 cells occur constitutively in the CNS like FoxP3 Tregs, but it is unlikely since they are generated from activated T cells during an immune response and have more of a suppressive than preventive role (Roncarolo et al., 2018).

Evidence therefore is emerging of a central role for CNS specific, resident, regulatory T cells and particularly FoxP3+ Tregs, as the agents of CNS IP which is taking its place as a major fine‐tuning mechanism of CNS IP alongside older dogma based on tissue barriers, and functionally may prove to be of greater impact.

## 8 TREGS CONTROL CNS INFECTION BY PROMOTING LATENCY

How do CNS‐specific Tregs control/prevent CNS infection? Bacterial infections in particular pose a problem. Entry to the CNS can occur directly or by ‘Trojan horse’ mechanism as cargo inside activated leukocytes crossing blood–brain or blood–retinal barriers. In addition, infectious agents may gain access from the nasal cavity to the brain via the cribriform plate and directly traffic to the brain along axons of the olfactory nerve and/or by infecting associated glial cells (reviewed in Forrester et al., 2018). We have argued previously that the immunoquiescent (‘privileged’) environment of the CNS parenchyma is conducive more than other tissues to the induction of latent infection (Forrester et al., 2018, 2022) and it does so by using Tregs to ‘tune down’ the activity of Teffs but also by directly acting on the latently infected cells. Tregs are heterogeneous and may be polarised similar to Teff cells. For instance, IFNγ Tbet‐ expressing CD8+ T cells are involved in HSV infection in brain, as is granzyme B, and both processes promote viral latency in trigeminal ganglion neurones (Knickelbein et al., 2008; St Leger & Hendricks, 2011). However, Tregs are known to be involved in promoting and/or sustaining HSV latency (Suvas et al., 2004). For instance, Yu et al. showed that HSV‐1 latency is abolished when Tregs are depleted and restored when Tregs are reconstituted (Yu et al., 2018). Interestingly, HSV lytic antigens persist in the ependyma following clearance of HSV from the brain, suggesting that location of viral infection influences the level of latency (Menendez et al., 2016).

Tregs are also involved in *Mycobacterium tuberculosis* infection. In latent CNS *M. tuberculosis* infection, the bacterium establishes a replicative niche inside macrophages, of which there are dense populations in the meninges and uveal tract (Dando et al., 2019; McMenamin, 1997) and are constantly under threat of inducing inflammation if detected by Teff cells. Higher levels of Treg versus Th17 cells promote extrapulmonary dissemination including to the brain (Cardona & Cardona, 2019) where local Tregs sustain latent infection and the balance between an overactive Teff response or relapse into caseation/abscess formation. In immunosuppressed patients (e.g. AIDS), the risk of the latter is increased. However, this is prevented by *M. tuberculosis*‐specific Tregs which control Teff cells (Sun et al., 2024).

A similar scenario plays out in *Toxoplasma* infection in the brain (Sasai & Yamamoto, 2019; Wohlfert et al., 2017) and even in less frequent infections such as cryptococcal infection (Alanio, 2020; Hill & Aguirre, 1994; Snarr et al., 2020). In CNS *Toxoplasma* infection, a common disease in immunocompromised patients (see above), Tbet expression by Tregs is required to prevent *T. gondii* bradyzoites (slow growing latent form encased within tissue cysts) from converting to fast‐replicating tachyzoites (Warunek et al., 2021) that cause acute toxoplasmosis.

Several other examples of immune cell regulation of latent CNS infection can be cited such as CMV (Almanan et al., 2017), ZIKA (Graham et al., 2024), Measles virus and West Nile Fever virus (Miller et al., 2019), where a similar pattern of disease induction, latency and reactivation regulated by the tissue immune response can be observed. Induction of latency might be envisaged as an attenuated CNS host response mediated by IP (Tregs) when full clearing of the pathogen would invoke an overly strong damaging immune response.

Tregs are likely generated locally in the CNS border tissues by their interaction with CD11c+ DC, presumably in the recently described Treg:DC clusters (Marin‐Rodero et al., 2025; O'Brien et al., 2017). We suggest that the particular microenvironment of the CNS epigenetically imprints its signals on the CNS Treg transcriptome to promote IP. In this way, organisms would normally be easily prevented from replicating and causing cytolysis, but when the Treg ‘brake’ is released and antigen‐specific Teffs are too scarce to prevent the microbe from reactivating, lethal cytolytic CNS inflammation would ensue.

## 9 HOW EFFECTIVE IS CNS IMMUNE PRIVILEGE (TREGS) IN REDUCING THE RISK OF CNS INFECTION?

CNS immune defences include innate and adaptive mechanisms. Immune defences can be subverted, for instance, when bacteria co‐opt the CGRP‐RAMP1 axis to invade the brain in bacterial meningitis (Pinho‐Ribeiro et al., 2023). Appropriate levels of Tregs to Teff are essential to control fungal infections (Dangarembizi et al., 2025) and to prevent *T. gondii* tachyzoite reactivation (Landrith et al., 2015). Similarly, an adequate Treg/Teff balance is required to prevent an exaggerated Teff response to herpes virus infection (Fu & Pan, 2025) with subsequent non‐pathogen‐related tissue damage. This immune imbalance can affect other immune cells as when there is insufficient homing of gut‐educated plasma cells to the meninges (Fitzpatrick et al., 2020) for defence against fungi. Indeed, a link between gut dysbiosis and neuroinflammation has recently been reported mediated by commensal antigen‐specific T cells which found their way from activation in the gut to the CNS (White et al., 2025).

Despite this evidence, the ubiquity of pathogens of all types, the high frequency of exposure and minimally symptomatic infection of large tracts of the world's populations and the relative infrequency of serious CNS infection all speak to a high level of control of CNS infection. This appears to be contingent on not only well‐recognised tissue‐vascular barriers, but also a local, powerfully protective immune regulatory system, which is transcriptionally distinct from that of the systemic immune regulatory system, or from immunoregulation in other organs, and is particular to the CNS, manifesting as ‘immune privilege’ described many years ago by Medawar and his colleagues (Medawar, 1948, 1961).

## 10 CONCLUSION

Despite widespread exposure to, and infection by, numerous microbes, which affect the CNS in many different geographical settings, the incidence of serious CNS infection is low, if not rare. Replicating pathogens are detected and cleared by the immune system from mucosal and skin sites of entry but some traffic to distant sites, including the CNS. CNS‐resident Tregs, located in and specific to the CNS, restrain the cytolytic anti‐pathogen immune response thus permitting pathogen survival, but instead promote latency. Because CNS Tregs control the Teff/cytolytic response, they can be regarded as the source of ‘immune privilege’, an immunoprotective function which they provide to the host in its interaction with CNS pathogens. CNS IP is, in essence, a combined form of tolerance, both immune tolerance and disease tolerance (Medzhitov et al., 2012; Wang et al., 2018). This precarious host–pathogen stand‐off is undermined when the host is immunocompromised and the pathogen reactivates, sometimes to cause fatal infection. Alternatively, the host might belatedly mount a classical immune response which, if exaggerated, can also cause severe pathology.

## FUNDING INFORMATION

Saving Sight in Grampian/Development Trust of University of Aberdeen: RG16220‐10.
